# Pretransplant Fasting Glucose Predicts New-Onset Diabetes after Liver Transplantation

**DOI:** 10.1155/2012/614781

**Published:** 2012-01-29

**Authors:** Elizabeth J. Carey, Bashar A. Aqel, Thomas J. Byrne, David D. Douglas, Jorge Rakela, Hugo E. Vargas, Adyr A. Moss, David C. Mulligan, K. Sudhakar Reddy, Harini A. Chakkera

**Affiliations:** ^1^Division of Hepatology, Mayo Clinic Arizona, 5777 E. Mayo Boulevard, Phoenix, AZ 85054, USA; ^2^Division of Transplant Surgery, Mayo Clinic Arizona, Phoenix, AZ 85054, USA; ^3^Division of Nephrology, Mayo Clinic Arizona, Phoenix, AZ 85054, USA

## Abstract

New-onset diabetes after transplantation (NODAT) is common after liver transplant and associated with poorer outcomes. The aim of this study was to identify risk factors for NODAT in liver transplant recipients off corticosteroids. In 225 adult nondiabetic liver transplant recipients, the mean age was 51.7 years, the majority were men (71%), and half had HCV (49%). The mean calculated MELD score at transplantation was 18.7, and 19% underwent living-donor transplant (LDLT). One year after transplantation, 17% developed NODAT, and an additional 16% had impaired fasting glucose. The incidence of NODAT in patients with HCV was 26%. In multivariate analysis, HCV, pretransplant FPG, and LDLT were significant. Each 10 mg/dL increase in pretransplant FPG was associated with a twofold increase in future development of NODAT. The incidence of NODAT after liver transplant in patients off corticosteroids is 17%. Risk factors for developing NODAT include HCV and pretransplant FPG; LDLT is protective.

## 1. Introduction

Liver transplant recipients are at risk of a number of metabolic complications including new-onset diabetes mellitus, hypertension, hyperlipidemia, and vascular disease. Many of these complications are caused or exacerbated by immunosuppression. As graft survival has improved, transplant recipients are increasingly affected by the long-term complications of these metabolic conditions. Long-term management of transplant recipients now highlights the importance of identifying and managing the metabolic complications of transplantation and immunosuppression [1]. 

New-onset diabetes after liver transplantation (NODAT) is an incompletely understood phenomenon estimated to occur in 15–30% of recipients who were not diabetic prior to transplant [2]. Diabetes mellitus is common in transplant recipients and is associated with increased rates of rejection, infection, cardiovascular disease, and with decreased survival [2–6]. However, there is a wide variation in the reported definition and incidence of NODAT due to heterogeneity of study design, variability of corticosteroid dose and immunosuppression protocol, and definition of posttransplant diabetes mellitus. Additionally, many cirrhotic patients have diabetes prior to transplantation, and not all of the reported literature excluded patients with preexisting diabetes mellitus [2].

Diabetes mellitus exerts a significant toll on the health of liver transplant recipients. It is associated with an increased rate of cardiovascular events, infectious diseases, chronic kidney disease, rejection, and with lower patient survival [3, 5, 7]. These factors, in conjunction with the financial burden of managing diabetes mellitus, provide compelling reasons to study the prevention of NODAT. Identifying patients at highest risk of NODAT will help researchers design clinical prevention trials.

The aims of this study were (1) to determine the incidence of NODAT using the American Diabetes Association (ADA) definition in a cohort of liver transplant recipients who were not diabetic prior to transplantation and who were weaned off corticosteroids after transplantation and (2) to determine pretransplant risk factors associated with NODAT in this cohort. 

## 2. Materials and Methods

### 2.1. Patients

This study was approved by the Mayo Clinic Institutional Review Board. All nondiabetic patients undergoing initial liver transplantation at Mayo Clinic Arizona were included. All patients had at least one year of followup. From June 1999 through February 2008, 425 liver transplants were performed. Two hundred patients were excluded for pretransplant diabetes mellitus (104), followup less than one year (72), retransplantation (19), dual organ transplantation (4), and other (1), leaving 225 patients in the study cohort. Patients on corticosteroids before transplantation and after the standard 4-month taper were excluded. 

Pretransplant variables collected include age, gender, race, etiology of liver disease, family history of diabetes mellitus, fasting plasma glucose (FPG), cholesterol, triglycerides, body mass index, and model for end-stage liver disease (MELD) score at the time of transplant. All MELD scores were reported as the calculated value, and MELD exception points were not included. The laboratory values were collected from the most recent outpatient visit prior to transplantation; the interval between laboratory collection and transplantation was less than 3 months in all patients. Donor variables collected included donor age, gender, race, and hepatitis C status. Hepatitis C recipients who were not viremic after transplantation, either due to spontaneous clearance or antiviral therapy, were categorized into the non-HCV group for analysis. At 1, 4, and 12 months after transplantation, the following variables were recorded: body mass index, FPG, immunosuppression, use of medication to control blood sugar, and prednisone dose. 

### 2.2. Immunosuppression

All patients followed a standard immunosuppression protocol using tacrolimus and mycophenolate mofetil. All patients received 1 gram of intravenous methylprednisolone on the day of transplant, followed by an oral course of prednisone which was tapered in a standard fashion with cessation at 4 months after transplant. In cases of neurotoxicity or other intolerance to tacrolimus, cyclosporine was substituted. Mycophenolate mofetil was adjusted or discontinued in cases of gastrointestinal side effects or myelosuppression. In rare cases of intolerance to calcineurin inhibitors, rapamycin was substituted for tacrolimus or cyclosporine. 

### 2.3. Definition of Diabetes Mellitus

NODAT was defined using the ADA definition of diabetes [8]. NODAT was assessed at one year after transplantation to exclude patients with transient hyperglycemia related to the stress of surgery and effect of corticosteroids.

ADA definition of diabetes mellitus [8]:

1. hemoglobin A1c ≥ 6.5%,
2. fasting blood glucose ≥ 126 mg/dL on 2 consecutive occasions,
3. random blood glucose ≥ 200 mg/dL,
4. blood glucose ≥ 200 mg/dL 2 hours after drinking a beverage containing 75 grams of glucose dissolved in water after an overnight fast.

Impaired fasting glucose was defined as a fasting plasma glucose level of 100 mg/dL to 125 mg/dL. 

### 2.4. Statistical Analysis

Continuous variables were described using mean, standard deviation, and *t*-tests. The chi-square test was used for description of categorical variables. Logistic regression was used to determine the risk associated with pretransplant patient characteristics on the development of NODAT. Unadjusted (univariate) logistic regression models were initially performed followed by multivariate analysis of variables that were statistically important in the univariate analysis. A *P* value of <0.05 was considered statistically significant.

## 3. Results

From June 1999 through February 2008, 225 nondiabetic patients underwent liver transplantation. Demographics of the study patients are listed in Tables 1 and 2. 

At one year after transplantation, the mean FPG was 106 mg/dL, and 8% were using insulin (no patients were on medications other than insulin for management of blood sugar). Thirty-six percent of patients were obese, and 10% were morbidly obese. At one year after transplant, 17% met the ADA definition of diabetes mellitus, and 16% had impaired fasting glucose (Table 3). Of patients with HCV, the incidence of NODAT was 26%.

Tacrolimus was the primary immunosuppressant in the majority (91%) of patients after transplantation with mycophenolate as an adjunctive agent and a prednisone taper as previously described. The NODAT+ and NODAT− groups showed no difference in tacrolimus levels, episodes of acute cellular rejection treated with intravenous steroids, or cumulative steroid dose.

**Figure 1:**
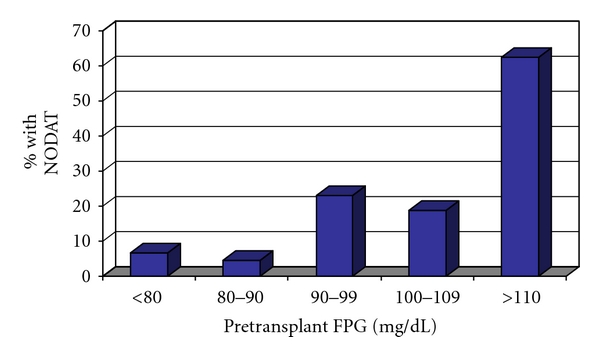
Incidence of NODAT by pretransplant fasting plasma glucose (*N* = 225).

Univariate analysis results demonstrate that male gender, presence of HCV, type of transplant (living-donor versus deceased donor), and pretransplant FPG were significantly associated with NODAT (Table 4). In multivariate analysis, HCV and pretransplant FPG were associated with NODAT, whereas living-donor liver transplantation appeared to be protective (Table 5). Each 10 mg/dL increase in the fasting plasma glucose was associated with 2-fold higher risk (OR 2.1 CI 1.37–3.31) of development of NODAT (Figure 1). There was no statistically significant association observed between patient age, ethnicity, family history of diabetes mellitus, pre- or posttransplant body mass index, cholesterol, or triglycerides and future NODAT. 

## 4. Discussion

In this study, using the ADA definition of diabetes mellitus, the incidence of NODAT one year after transplantation in a steroid-free cohort of liver transplant recipients was 17%, and an additional 16% had impaired fasting glucose. Abnormal glucose homeostasis thus occurred in 33% of a cohort of liver transplant recipients on a standardized regimen of immunosuppression with tacrolimus, mycophenolate mofetil, and a rapid steroid taper completing at 4 months. Independent predictors for NODAT include the pretransplant fasting plasma glucose, the presence of hepatitis C virus, and the type of transplant (living-donor versus deceased donor). 

In the general population, fasting plasma glucose predicts the future development of type 2 diabetes mellitus, even when the FPG is within the normal range [9]. Pretransplant FPG has also been shown to predict NODAT in kidney transplant recipients [10]. This effect occurs not only in patients with impaired fasting glucose, but also in those with FPG < 100 mg/dL. In this way, NODAT appears to be similar to type 2 diabetes mellitus although additional research is needed to determine if the pathophysiology is the same.

Hepatitis C virus is a substantial and consistent risk factor for the development of diabetes mellitus both before and after liver transplantation [11]. In line with current literature, this study found hepatitis C virus to be a significant risk factor for development of NODAT, with an odds ratio of 3.7 (CI 1.64–8.36). Prior studies have demonstrated a correlation between insulin resistance and HCV viral load [12], and fewer glucose abnormalities in sustained virologic responders after antiviral therapy [13]. Another independent risk factor was the pretransplant fasting plasma glucose, with every 10 mg/dL increase in the pretransplant fasting plasma glucose resulting in a 2-fold increased risk of NODAT. This is the first report of a relationship between the pretransplant FPG and the development of NODAT in a cohort of liver transplant patients off corticosteroids. 

Living-donor transplantation was noted as a protective factor for the development of NODAT in one previous paper [14], but the reasons remain unclear. In our study, the deceased donor recipients had a higher calculated MELD score than the living-donor recipients, indicating more advanced disease in the deceased donor group. Glucose homeostasis is a complex process requiring interplay between multiple hormones and metabolic mediators and their effects on liver, skeletal, and adipose tissues. Chronic liver disease, even in the absence of HCV, is associated with altered glucose tolerance. Glucose metabolism is significantly impaired in patients with chronic liver disease and worsens with more advanced liver disease [15]. The higher incidence of NODAT noted in deceased donor recipients may be a result of more inflammation and more advanced liver disease as compared to the living-donor recipients. Patient and graft survival of living-donor liver transplant recipients has recently surpassed that of deceased-donor liver transplant recipients [16]. Whether a relationship exists between the development of NODAT and outcomes after living-donor liver transplantation has not been studied, and future research will address this question further. 

Another intriguing outcome of this study was the finding that, in univariate analysis, donor Hispanic race was associated with risk for NODAT. Hispanic race is a known risk factor for type 2 diabetes mellitus in the general population [17]. Race has been shown to be related to NODAT after liver transplantation for African-American race but not for Hispanic race [14]. The presence of diabetes in the donor is also related to an increased risk of NODAT after liver transplantation. Considering that Hispanic donors may be more likely to have diabetes than Caucasian donors, and that donor diabetes increases the risk of NODAT, it is possible that the increase in NODAT seen in recipients who received an organ from a Hispanic donor may simply be due to a higher prevalence of diabetes in this donor pool. The retrospective design of this study is a limitation which precludes further analysis of this observation.

The incidence of NODAT after liver transplantation ranges from 18 to 36% in the literature [18, 19]. In this series, 17% of nondiabetic liver transplant recipients developed NODAT at one year after transplantation. The range in incidence is likely due to differences in study design and evolution of immunosuppression management. For example, older literature defined NODAT with a fasting plasma glucose > 140 mg/dL [4] and did not exclude patients with pretransplant diabetes [3] or those on corticosteroids [4, 19]. With the ADA criteria, we used a strict definition of NODAT and delayed the diagnosis of diabetes until one year after transplantation when all patients had been off corticosteroids for 8 months. 

Immunosuppression contributes to NODAT. Corticosteroids are known to be diabetogenic, likely due to the development of insulin resistance. The use of corticosteroids has been associated with NODAT, both when given as a bolus administration for management of acute cellular rejection [3] and as the cumulative steroid dose [19]. Both tacrolimus and cyclosporine are known to be diabetogenic with tacrolimus exerting a more substantial effect [20]. In this cohort on a standardized and homogenous immunosuppression protocol, we could not evaluate the effect of specific immunosuppressive agents on development of NODAT. 

The strengths of this study include a large sample size, the use of a modern cohort of liver transplant recipients, use of the ADA definition for diagnosis of NODAT, and a standardized immunosuppression protocol. Limitations include the retrospective study design and the single-center cohort; thus, these results may not be applicable to other transplant groups.

In summary, abnormal glucose homeostasis occurs in 33% of liver transplant recipients who were not diabetic prior to transplantation: NODAT develops in 17% and an additional 16% of patients develop impaired fasting glucose in the same time period. Pretransplant risk factors for the development of NODAT include hepatitis C virus and higher pretransplant fasting plasma glucose; living-donor transplantation appears to be protective. Given the prevalence of abnormal glucose homeostasis in liver transplant recipients, and its implication on long-term allograft and patient survival, future study to risk stratify patients and conduct preventive strategies is important.

## Figures and Tables

**Table 1:** Pretransplant demographics by development of NODAT.

	NODAT−	NODAT+	Total	*P* value
	*N* = 186	*N* = 39	*N* = 225	
Recipient age, years	51.7 ± 10.4	51.2 ± 5.9	51.7 ± 9.8	0.76
Male gender, *N* (%)	127 (68.3)	33 (84.6)	160 (71.1)	0.04
Recipient race, *N* (%)				0.83
Caucasian	140 (75.2)	30 (76.9)	170 (76.6)	
Hispanic	31 (16.7)	6 (15.4)	37 (16.4)	
Other	12 (6.5)	2 (5.6)	14 (6.3)	
Family history of DM, *N* (%)	58 (31.7)	16 (43.2)	74 (33.6)	0.17
Etiology of liver disease				0.001
HCV, *N* (%)	82 (44.1)	29 (74.3)	111 (49.3)	
ALD, *N* (%)	29 (15.6)	2 (5.1)	31 (13.8)	
Other	75 (40.3)	8 (20.5)	83 (36.9)	
LDLT, *N* (%)	41 (22.0)	2 (5.1)	43 (19.1)	0.02
Calculated MELD	18.7 ± 8.5	18.5 ± 7.9	18.7 ± 8.4	0.88
BMI	27.8 ± 5.2	29.2 ± 5.1	28.1 ± 5.2	0.13
Obese (BMI ≥ 30)	71 (38.1)	17 (43.6)	88 (39.1)	0.4
Morbid obesity (BMI ≥ 40)	4 (2.1)	1 (2.6)	5 (2.2)	0.02
Pre-tx FPG (mg/dL)	91 ± 8	97 ± 10	92 ± 9	0.0001
Pre-tx IFG *N* (%)	16 (8.6)	8 (20.5)	24 (10.7)	0.03
Pre-tx cholesterol (mg/dL)	135 ± 107	113 ± 44	132 ± 99	0.21
Pre-tx triglycerides (mg/dL)	89 ± 51	92 ± 49	89 ± 51	0.74
*Donor demographics*				
Male gender, *N* (%)	109 (58.6)	27 (69.2)	136 (60.4)	0.22
BMI, mean	26 ± 5.0	26.8 ± 5.7	26.1 ± 5.1	0.41
Age, years	40.1 ± 16.4	37.9 ± 16.3	39.7 ± 16.3	0.44
Hispanic race, *N *(%)	36 (19.3)	14 (35.9)	50 (22.2)	0.02

Continuous variables presented as means with standard deviations.

ALD: alcoholic liver disease, BMI: body mass index, DM: diabetes mellitus, FPG : fasting plasma glucose, HCV: hepatitis C virus, IFG: impaired fasting glucose, LDLT: living-donor liver transplantation, MELD: model for end-stage liver disease scores.

**Table 2:** Pretransplant demographics of living-donor and deceased-donor recipients.

	LDLT	DDLT	*P* value
	*N* = 43	*N* = 182	
Recipient age, years	49.1 ± 10.6	52.3 ± 9.5	0.06
Male gender, *N* (%)	26 (60.4)	134 (73.6)	0.09
Recipient race, *N* (%)			
Caucasian	31 (72.1)	139 (76.4)	0.56
Hispanic	9 (20.9)	28 (15.4)	0.39
Family history of DM, *N* (%)	10 (23.3)	64 (35.2)	0.13
HCV, *N* (%)	19 (44.2)	92 (50.5)	0.56
Calculated MELD	14.2 ± 5.8	19.7 ± 8.6	0.0001
BMI	26.3 ± 4.6	28.5 ± 5.2	0.01
Pre-tx FPG (mg/dL)	90.2 ± 6.7	92.3 ± 9.2	0.15
Pre-tx cholesterol (mg/dL)	158.6 ± 68.5	125.2 ± 104.6	0.05
Pre-tx triglycerides (mg/dL)	85.4 ± 37.8	90.4 ± 53.6	0.57

LDLT: living-donor liver transplant.

DDLT: deceased-donor liver transplants.

**Table 3:** Posttransplant variables by development of NODAT.

	NODAT−	NODAT+	Total	*P* value
	*N* = 186	*N* = 39	*N* = 225	
*One year*				
FPG (mg/dL)	98 ± 11.5	148 ± 50	106 ± 30	—
Hgb A1C	5.4 ± 0.3	6.3 ± 1.3	5.6 ± 0.7	—
BMI	27.1 ± 5.4	29.0 ± 6.9	27.5 ± 5.8	0.07
Tacrolimus level (ng/mL)	10.8 ± 3.2	11.3 ± 3.3	10.9 ± 3.2	0.4
Primary IS, *N* (%)				
Tacrolimus	170 (91.4)	33 (84.6)	203 (90.2)	0.74
Cyclosporine	6 (3.2)	2 (5.1)	8 (3.6)	
Sirolimus	10 (5.4)	3 (7.7)	13 (5.8)	
Other	0	1 (2.6)	1 (0.4)	
Steroid-treated rejection	0.2 ±.48	0.2 ±.47	0.2 ±.48	0.75
Cumulative steroid dose, mg	2811 ± 1447	2732 ± 1407	2797 ± 1437	0.75
*NODAT*			39 (17.3)	
*IFG*, *N* (%)	36 (16)			
*Total abnormal glucose homeostasis, N* (%)			75 (33.3)	

Continuous variables presented as means with standard deviation.

BMI: body mass index, FPG: fasting plasma glucose, Hgb A1C: Hemoglobin A1C, IFG: impaired fasting glucose, IS: immunosuppression, NODAT: new-onset diabetes after transplantation.

**Table 4:** Pretransplant predictors of NODAT in univariate analysis.

Predictor	DM at 1 year 39/225 = 17%	
	OR (CI)	*P*
Age per 1 year	0.99 (.9–1.0 )	0.8
Male	2.6 (1.0–6.4)	0.05
Caucasian race	1.1 (.5–2.5)	0.8
Hispanic race	0.9 (.35–2.2)	0.8
Family history of DM	1.5 (.7–3.1)	0.2
HCV	3.7 (1.7–7.9)	0.001
BMI per 1 unit	1.05 (.9–1.1)	0.13
FPG per 10 mg/dL	2.2 (1.4–3.4)	0.0001
Cholesterol per 1 mg/dL	0.99 (.99–1.0)	0.16
Triglycerides per 1 mg/dL	1.0 (.99–1.0 )	0.7
LDLT	0.2 (.04–.83)	0.03

Age > 50	0.6 (.3–1.2)	0.13
FPG > 100 mg/dL	4.6 (1.5–14.7)	0.009
Cholesterol > 200 mg/dL	0.7 (.2–2.6)	0.6
Triglycerides > 200 mg/dL	1.5 (.47–4.9)	0.5
BMI > 30	1.2 (.6–2.4)	0.7

**Table 5:** Pretransplant predictors of NODAT in multivariate analysis.

Predictor	OR (CI)	*P*
FPG per 10 mg/dL	2.1 (1.37–3.31)	.001
LDLT	.22 (0.05–0.98)	.05
HCV	3.7 (1.64–8.36)	.002

## List of Abberviations

NODAT: New-onset diabetes after transplant
ADA: American Diabetes Association
FPG: Fasting plasma glucose
MELD: Model for end-stage liver disease score
HCV: Hepatitis C virus.
